# Pathogenic mechanisms in Fabry disease

**DOI:** 10.3389/fmed.2026.1867822

**Published:** 2026-06-23

**Authors:** Siming Wang, Chengyue Sun

**Affiliations:** Department of Neurology, Peking University People's Hospital, Beijing, China

**Keywords:** Fabry disease, Gb3 accumulation, lysosomal storage disorder, multi-system involvement, α-galactosidase A

## Abstract

Anderson-Fabry disease (FD) is a rare X-linked lysosomal storage disorder caused by deficient activity of the enzyme α-galactosidase A, resulting in progressive accumulation of glycosphingolipids, particularly globotriaosylceramide (Gb3), across multiple organs. FD exhibits marked phenotypic variability influenced by residual enzyme activity, mutation type, and X-chromosome inactivation. Pathogenic mechanisms involve genetic, enzymatic, and metabolic dysregulation, leading to lysosomal dysfunction, inflammation, oxidative stress, mitochondrial impairment, and autophagic defects. Multi-system involvement includes vasculopathy, cardiovascular abnormalities such as left ventricular hypertrophy and arrhythmias, progressive renal injury, central nervous system lesions with cognitive and psychiatric manifestations, and peripheral neuropathy characterized by pain and autonomic dysfunction. Accumulation of Gb3 and its derivative globotriaosylsphingosine (lyso-Gb3) underlies both direct cellular toxicity and secondary pathological processes, including inflammatory activation, oxidative damage, and ion-channel dysregulation. This review synthesizes current understanding of the molecular and cellular pathogenesis of FD, highlights mechanisms underlying phenotypic variability, and integrates organ-specific manifestations.

## 1 Introduction

Anderson-Fabry disease (FD) (OMIM #301500) is a rare, X-linked, inherited lysosomal storage disorder (1). It is caused by partial or total deficiency of the enzyme α-galactosidase A (2). This enzymatic defect results in chronic intracellular accumulation of glycosphingolipids, particularly globotriaosylceramide (Gb3) (3), in the tissues and organs of the whole body, leads to multi-system involvement. The galactosidase α (GLA) gene is located on the long arm of chromosome X (Xq22.1) and consists of 7 exons, encoding the α-galactosidase A protein (4). The incidence in the general population is estimated to range from 1 in 40,000 to 1 in 117,000 (5). In classically affected individuals, progressive glycosphingolipid accumulation, particularly in the vascular endothelium, leads to manifestations including angiokeratoma, acroparesthesia, gastrointestinal symptoms, and corneal opacities during childhood or adolescence. With advancing age, the progressive vascular involvement results in renal insufficiency, cardiac disease, and strokes (6). Due to the development of a specific vasculopathy, patients with FD have a shortened life expectancy. Typically, male patients develop renal impairment in their third or fourth decade of life, as well as cardiovascular abnormalities. Median life expectancy in the male population is reduced to 52–62 years (7). In females, the disease is more variable, since females can be affected from a milder to a severer degree because of random X-chromosomal inactivation (8). In addition, endoplasmic reticulum (ER) stress and the activation of unfolded protein response (UPR) induced by misfolded α-galactosidase A (α-Gal A) may also contribute to disease manifestations in some heterozygous females despite normal or only mildly decreased α-Gal A activity (9). Female patients often have less renal involvement, but life span is shortened as well, with a median of 69 to 75 years in the female population (7). On average, life expectancy is estimated to be reduced by 15 years in females (10) and 20 years in males (11).

In this review, we first discuss phenotypic variability in FD, including the effects of residual enzyme activity and X-chromosome inactivation. Subsequently, we analyze and discuss the molecular and cellular pathogenesis of FD based on recent research. In the end, we analyze and discuss pathogenic mechanisms in relation to five major affected organs/systems: blood vessels, heart, kidneys, central nervous system, and peripheral nervous system.

## 2 Phenotypic variability

Patients are generally classified into two major groups depending on whether residual α-Gal A activity is detectable: a classic phenotype and atypical variants characterized by milder symptoms and later onset (12).

In classic form of FD, males often exhibit undetectable enzyme activity and develop disease signs and report symptoms much earlier than females with classic FD (13). In males with FD, symptoms appear in the first decade of life, typically with multi-organ involvement. Primarily, this phenotype involves kidneys and/or heart (referred to as “renal variants” or “cardiac variants”) (14). There are also male patients who have a late-onset disease, with symptoms appear in the third to the sixth decade of life. In this phenotype, patients exhibit some residual enzyme activity (up to 10% of normal) (15). Several studies have shown that, compared with patients harboring other mutations, people carrying the N215S mutation generally have a milder phenotype with less organ involvement and later onset (16, 17).

In contrast to most X-linked disorders, a substantial proportion of female heterozygotes with FD exhibit symptoms, and in some cases may be affected as severely as males, although disease progression is generally slower (10). In females, the phenotypic variability of FD is strongly modulated by the pattern and extent of X-chromosome inactivation (XCI). XCI has historically been proposed as a key mechanism underlying the penetrance and expressivity of X-linked diseases in heterozygous females. Echevarria et al. (2016) reported that, α-Gal enzyme activity variability in heterozygous patients is strongly correlated with XCI (18). Due to XCI, heterozygous females with X-linked disease are mosaics, with different cells in the body expressing either the normal or disease allele in an approximately 1:1 ratio (19). Usually, this expressing pattern is sufficient to spare heterozygotes females from the clinical manifestations of the disease (18). Nevertheless, numerous studies have reported that heterozygous females may exhibit severe symptoms, suggesting that mechanisms such as skewed X-inactivation and allele-specific DNA methylation may also play a critical role in disease expression (20).

Recent studies have indicated that ER stress and UPR activation induced by misfolded α-Gal A proteins may also contribute to phenotypic variability in FD. Since intracellular retention and abnormal processing of mutant α-Gal A can trigger chronic ER stress independently of lysosomal substrate accumulation, pathological changes may persist even in the presence of relatively preserved enzymatic activity. This mechanism may therefore contribute to the marked phenotypic variability observed among heterozygous female FD patients (9).

Moreover, accumulating evidence indicates that identical mutations can manifest with variable phenotypes even within the same family, whereas different mutations may produce similar clinical outcomes, underscoring the absence of a clear genotype–phenotype correlation in FD (21). It has been proposed that pathological accumulation of globotriaosylsphingosine (lyso-Gb3) in FD patients may activate different cellular pathological processes (such as autophagy dysfunction and inflammation), contributing to intra-familial phenotypic variability (22). Several studies have also shown that non-genetic and epigenetic factors, such as dietary and environmental influences, may also contribute to phenotypic variability (23).

## 3 Genetic basis of FD

### 3.1 Mutations

FD is an X-linked lysosomal storage disorder secondary to mutations in the *GLA* gene located on the X chromosome. The *GLA* gene is a 14-kb gene located on Xq22.1, encodes α-Gal A (EC 3.2.1.22; Uniprot P06280) (24). To date, more than 1,100 mutations of *GLA* gene have been identified (reported in the Human Gene Mutation Database.

http://www.hgmd.cf.ac.uk/ac/gene.php?gene=GLA, accessed on September 5, 2025). The majority of mutations are private, occurring in only one or a few families. Among variants listed in the Human Gene Mutation Database, more than 70% of disease alleles are caused by point mutations, primarily missense [approximately 69% (4)] or nonsense variants. Small deletions or insertions contribute to approximately 25% of reported alleles, whereas large deletions or insertions (up to ten nucleotides) and complex mutations are rare. Pathogenic variants have been identified across all exons of the gene (25).

Mutations in the *GLA* gene can exert pathogenic effects through multiple mechanisms. While many mutations affect α-galactosidase A through amino acid substitutions or truncating mutations, some have been shown to disrupt mRNA processing, which in turn influences transcription and translation process. According to Lai et al. (2003), certain point mutations, although initially predicted to be deleterious due to amino acid substitutions, exerted their pathogenic effect by disrupting evolutionarily conserved splice consensus sequences, leading to abnormal mRNA splicing (26). Moreover, allele-specific DNA methylation at the promoter of the *GLA* gene may determine the expression level of mutated genes, thereby affecting the onset and progression of FD. DNA methylation is an epigenetic modification that primarily targets cytosines at CpG sites, occurs on both X-linked and autosomal genes and plays a key role in regulating gene expression (27).

### 3.2 Inheritance

For male patients, they inherit the mutation exclusively from their mothers, as the GLA gene is located on the X chromosome. Thus, they are always hemizygous. Accordingly, they can pass on the mutated *GLA* gene only to their daughters (28).

For female patients, although they can have mutated *GLA* gene on both X-chromosomes, the homozygous form is extremely rare. In females, *GLA* mutations are typically present in the heterozygous state (29). In heterozygous females, each offspring—whether male or female—has a 50% risk of inheriting the mutated gene (30).

Unlike traditional X-linked recessive hereditary disease, females who carry the mutated gene may also exhibit a diverse range of signs and symptoms of the disease, sometimes their level of clinical severity is equivalent to males (31). Thus, the definition of “carrier”, which refers to “An individual with a recessive pathogenic variant at a particular locus on one chromosome of a pair who is not expected to develop manifestations of the related condition” (32), is no longer accurate in describing heterozygous females. Now FD is regarded as an “X-linked transmission” disease (33). The Lyon hypothesis, or random X-inactivation, has been proposed to explain the phenotypic expression of FD in heterozygous women. Additionally, skewed X-inactivation may underlie the occurrence of severe clinical manifestations in heterozygous females (15).

## 4 Molecular defects of α-Gal A

α-Gal A is a lysosomal enzyme composed of approximately 429 amino acids and is responsible for catalyzing the hydrolysis of globotriaosylceramide (Gb3) into galactose and lactosylceramide (34). Deficient α-Gal A activity leads to the progressive accumulation of glycosphingolipids, predominantly Gb3, within the lysosomes of vascular endothelial cells.

**Figure 1:**
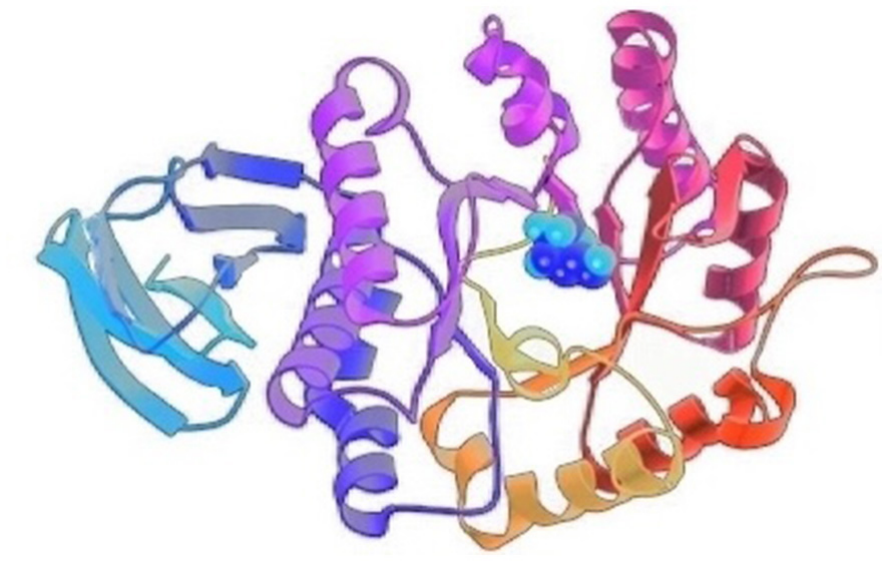
The structure of α-Gal A.

α-Gal A is a homodimeric glycoprotein consisting of two domains, a C-terminal domain and a (β/α)_8_ barrel domain [Figure 1].

**Figure 2:**
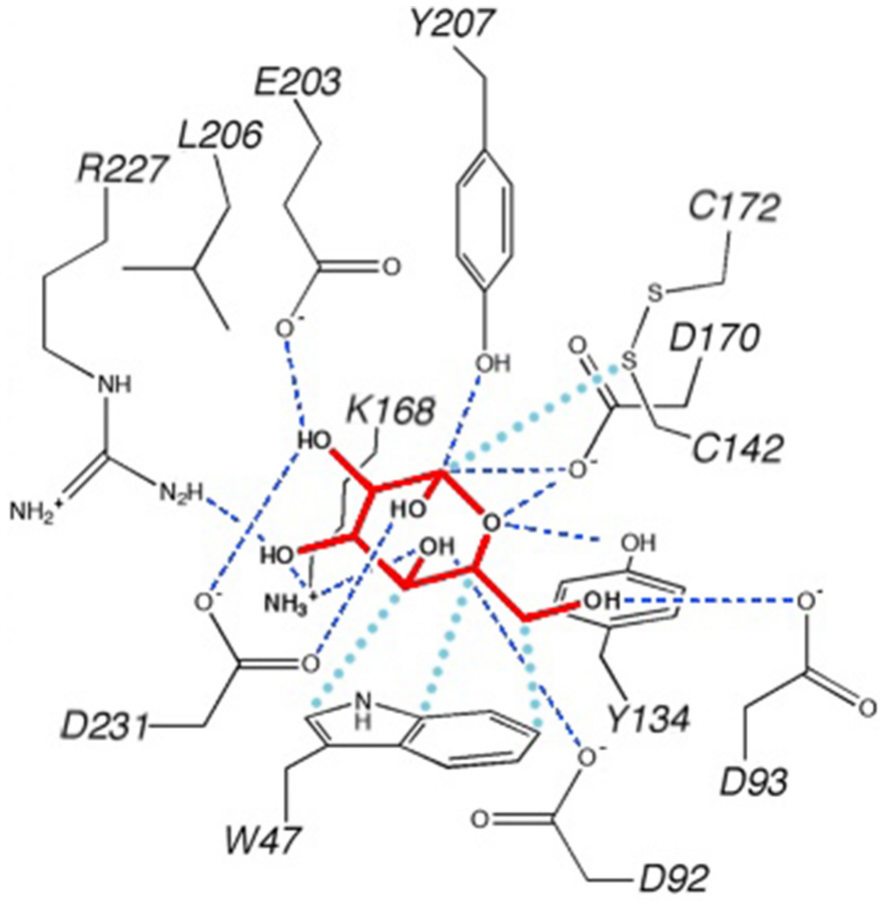
Active site of α-Gal A.

The C-terminal domain characterized by a β-sandwich architecture formed by two sheets of eight antiparallel β-strands. The (β/α)_8_ domain harboring the active site, which is composed of the side-chains of residues W47, D92, D93, Y134, C142, K168, D170, E203, L206, Y207, R227, D231, D266, and M267, with a disulfide bond between C172 and C142 [Figure 2]. Beyond these 13 critical residues, the remaining amino acid residues in a-Gal A primarily contribute to maintaining the tertiary structure.

Structural studies revealed that mutations in any of the 13 active-site amino acid residues lead to the severe classic phenotype (35–37). According to Tsukimura et al. (2014), the mutation, C142Y, has been suggested to prevent a galactose residue from entering the active pocket. This mutation induces only moderate conformational changes in the enzyme structure. Although the mutant α-Gal A with C142Y may lose enzyme activity completely, the translated α-Gal A protein may still partially escape degradation and reaches the lysosomes, which is consistent with the classic phenotype observed in patients (38). It has also been reported that, even when the C142Y mutant *GLA* is expressed at half the normal level, the catalytic activity may be completely lost (39). Not only mutations of active-site amino acid residues, but also other mutations, such as E59K, A156V, L166V, R356W, and G373S, have been identified in classic FD patients (39–41).

The structural analysis also revealed that R112H induces a relatively large structural alteration on the protein surface, whereas M296I causes a minor change in a localized region extending from the core to the surface; both mutations are located far from the active site. In these cases, folding defects and partial degradation of the enzyme, along with residual structural activity, contribute to disease pathogenesis. The remaining α-Gal A protein can partially degrade plasma lyso-Gb3, but this is insufficient for complete substrate clearance in organs, leading to a late-onset phenotype (38).

Studies have also looked into the mechanisms underlying decreased enzyme activity. Ishii et al. (2007) demonstrated that, in patients with residual enzyme activity, deficiencies may be due to abnormal processing and trafficking or altered kinetic activity (42).

Excessive degradation of mutant protein or their combinations can also contribute to the loss of enzyme activity. Protein misfolding has been recognized as an important pathophysiological cause (43). Mutant proteins with misfolded conformations are recognized by the endoplasmic reticulum (ER) quality-control system and subsequently degraded via the ER-associated degradation pathway (44). According to Fan et al. (2007), excessive degradation of mutant α-Gal A can lead to reduced enzymatic activity (45).

## 5 Molecular and cellular pathogenesis of FD

α-Gal A is responsible for hydrolyzing terminal α-galactosyl residues from Gb3 and related glycosphingolipids, constituting a critical step in their normal catabolic pathway (46). Mutant α-Gal A results in deficiency of enzymatic activity, which in turn leads to progressive lysosomal accumulation, leads to lysosomal storage disorders (LSDs). The pathological storage of Gb3 and its derivative lyso-Gb3 promotes the deposition of myelin-like inclusions within lysosomes, a hallmark of FD pathology (47). This pathological accumulation induces lysosomal dysfunction, which in turn perturbs multiple intracellular signaling pathways and contributes to progressive cellular dysfunction and organ damage (48).

Lysosomal storage disorders (LSDs) comprise a group of more than 50 monogenic diseases caused by defects in proteins essential for lysosomal metabolism (49). Lysosomes are membrane-bound acidic organelles present in most animal cells and harbor over 60 hydrolytic enzymes responsible for macromolecule degradation. Impaired activity of these enzymes results in the progressive accumulation of undegraded substrates within lysosomes (50). FD is one such LSD, arising from deficient activity of α-Gal A. Ballabio et al. (2009) proposed that anomalous storage in lysosomes could affect signal transduction pathways, ultimately leading to cellular dysfunction (22).

### 5.1 Mitochondrial dysfunction

Mitochondrial dysfunction has been recognized as a critical factor in the pathophysiology of LSDs. Elsaid et al. (2023) reported that mitochondrial-related proteins are downregulated in FD animal models and FD patients, and energy-related pathways, including carbon metabolism, glycolysis, and galactose metabolism, are disturbed, suggesting a reduction in mitochondrial respiration and ATP production, impairing cellular energy supply (51, 52). A down-regulation of superoxide dismutase 2 activity was also confirmed in FD (51). Those alterations can impair lysosomal function through the generation of oxygen species (ROS) and by reducing ATP availability, which is essential for maintaining the acidic environment of autophagic vacuoles via the vacuolar-type ATPase (V-ATPase) proton pump (53).

Mammalian target of rapamycin (mTOR) plays a pivotal role in regulating mitochondrial metabolism and biogenesis through the promotion of nuclear-encoded gene translation (54). Liebau et al. (2010) demonstrated that Gb3 accumulation in Fabry podocyte cell cultures leads to a loss of mTOR kinase activity, suggesting that the buildup of metabolic substrates in lysosomes may disrupt the mTOR activation/inactivation cycle (55). Dysregulation of the mTOR signaling pathway can destabilize mitochondria and impair mitophagy, contributing to mitochondrial dysfunction (53).

Mitochondria DNA (mtDNA) is a double-stranded, closed-circle molecule that lacks histones. It encodes 37 genes, including 13 components of the mitochondrial electron transport chain (56). Simoncini et al. (2016) found a link between mtDNA sequence variations and FD phenotypes, indicating mtDNA may also contribute to the pathogenesis of FD (57).

Moreover, microRNAs (miRNAs) produced by mitochondrial genome may also influence FD at the molecular level. Mitochondrial miRNAs (mitomiRs) play a key role in the mitochondrial phenotype and are responsible for maintaining mitochondrial homeostasis (58, 59). Gambardella et al. (2023) discovered that mitomiRs are dysregulated in FD patients, suggesting that mitochondrial miRNAs may contribute to the pathogenesis of FD and can serve as biomarkers of FD (60).

As demonstrated by studies on various lysosomal storage disorders, mitochondrial physiology is closely related to morphology (61). Studies have demonstrated mitochondrial structural alterations in FD. According to Schumann et al. (2020) and Maruyama et al. (2018), mitochondria in FD cells appear shortened and swollen, frequently cluster around the nucleus, and exhibit disrupted cristae structure (52, 62). This alteration is accompanied by uncoupling of mitochondrial oxidative phosphorylation (OXPHOS), leading to increased ROS production, which consequently damages intracellular organelles and impairs cellular homeostasis (63). This suggests that a compromised mitochondrial energy supply via OXPHOS may contribute to the pathogenesis of organ dysfunction, particularly in tissues with high metabolic demands such as the heart and nervous system (64). Tricarboxylic acid (TCA) cycle, which functions as a central regulatory pathway in mitochondrial energy metabolism, integrating both anabolic and catabolic processes, is also disrupted in FD. Studies have revealed accumulations of TCA cycle metabolites (62), indicating altered substrate utilization within the mitochondrial TCA cycle. The accumulation of TCA cycle metabolites, particularly succinate (65), may further promote inflammatory reactions.

### 5.2 Inflammation

Pathological lysosomal deposits may function as damage-associated molecular patterns (DAMPs) or induce injured cells to release DAMPs. These processes subsequently initiate a pro-inflammatory cascade that contributes to damage of target organs (such as heart, kidney, and vasculature) (65).

Elevated levels of pro-inflammatory cytokines, including interleukin-6 (IL-6) and tumor necrosis factor-alpha (TNF-α), have been reported in FD patients. In addition, flow cytometry analyses demonstrated alterations in immune cell populations, suggesting an underlying immune dysregulation (66). Moreover, leukocytes and endothelial cells demonstrate inflammatory activation, characterized by abnormal immune cell counts and surface marker expression. Rozenfeld et al. (2009) reported abnormalities in the numbers of lymphocytes—including CD8+ T cells and B cells—as well as in monocytes and dendritic cells. They also observed altered expression of CD1d, major histocompatibility complex (MHC) class II, and CD31 on lymphocytes and monocytes in FD patients (67).

Recent progress in FD research has highlighted the pivotal contribution of inflammation to disease development, as well as the growing importance of emerging biomarkers in monitoring its course (68). Several key inflammatory biomarkers and mediators are summarized in Supplemental Table 1.

### 5.3 Oxidative stress

Accumulation of Gb3 and inflammation may trigger secondary biochemical processes that promote oxidative stress (69). Gb3 accumulation induces a dose-dependent increase in intracellular oxygen species (ROS) production, and upregulates adhesion molecule expression, including intercellular adhesion molecule-1 (ICAM-1), vascular cell adhesion molecule-1 (VCAM-1) and E-selectin, in vascular endothelial cells (63). Inflammatory activation further contributes to oxidative stress by enhancing ROS production (66). In addition, defective mitophagosome formation in FD may impair mitophagy, exacerbating oxidative stress (51). Excessive ROS can damage DNA, proteins, and lipids, ultimately leading to cellular dysfunction and contributing to the pathogenesis of FD (70). Previous studies have reported that FD patients with cardiomyopathy exhibit elevated levels of 8-hydroxy-2-deoxyguanosine (8-OHdG), a common by-product of DNA oxidation and a non-invasive marker of oxidative stress, in both serum and myocardial tissue (71–73). DNA damage in these patients appears to be persistent and is hardly fully repaired by endogenous repair mechanisms, ultimately leading to cellular dysfunction (74).

An altered antioxidant activity in FD has also been observed. Biancini et al. (2012) reported decreased levels of antioxidant defenses (erythrocyte glutathione (GSH) content and glutathione peroxidase (GPx) activity) in FD patients, indicating that hydrogen peroxide (H2O2) may be more prone to oxidize biological molecules in Fabry patients (66). They also found elevated levels of malondialdehyde (MDA) and protein carbonyls in patients' plasma, suggesting increased lipid peroxidation and protein oxidative damage. The continued oxidation and fragmentation of fatty acid side chains generate aldehydes, such as MDA (derived from the peroxidation of linolenic, arachidonic, or docosahexaenoic acids). These reactive aldehydes can disrupt lysosomal and other organelle membranes, readily bind to membrane proteins—thereby inactivating enzymes and receptors—and interact with DNA, leading to mutagenic lesions (75, 76).

Studies also suggested that Gb3 buildup in endothelium tissue may lead to nitric oxide (NO) dysregulation. Studies have suggested that pathological Gb3 accumulation may result in dysregulated activity of the endothelial nitric oxide synthase (eNOS), while the expression of the inducible NOS (iNOS) is upregulated (77, 78). iNOS produces higher levels of NO than eNOS. This excess NO readily reacts with other free radicals to produce reactive nitrogen species (RNS), leading to damage to surrounding macromolecules (79).

### 5.4 Autophagic dysfunction

Autophagy is a key process for maintaining cellular homeostasis, involving the degradation of cytoplasmic materials in the lysosome. It functions both to eliminate damaged components and to recycle essential building blocks and energy, thereby supporting cellular renewal and stress response (80). Gb3 accumulation in lysosomes results in an increase in lysosomal pH, mediating lysosomal destabilization and impairing lysosomal function (53).

Multiple studies have reported autophagic dysfunction in FD patients, and it has emerged as a critical mechanism in FD pathogenesis (81–83). mTOR alternates between activation and inactivation states to regulate the autophagosome-lysosome fusion process. Failure of mTOR reactivation results in the inhibition of autophagy (84). Studies have demonstrated that Gb3 accumulation leads to reduced mTOR kinase activity and impaired autophagy in podocytes and fibroblasts from FD patients (55, 81). These findings suggest that the dysregulation of mTOR signaling in FD contributes to autophagic dysfunction.

Autophagic dysfunction is involved in multiple pathogenic mechanisms underlying FD, contributing to the progression of organ involvement across multiple systems. Autophagy defects can lead to the accumulation of abnormal substances within tissues, which are thought to contribute to the pathogenesis of various cardiovascular conditions (85). In FD, one of the key manifestations of organ damage is ischemic stroke. Studies in FD patients have demonstrated that vascular smooth muscle cell (VSMC) proliferation occurs in cerebral arteries, establishing a link between autophagic dysfunction and small vessel disease (85, 86). Furthermore, in animal models, widespread neurological pathology, including axonal neurodegeneration, has been observed in α-Gal A-deficient mice. These changes are associated with disruption of the autophagy-lysosome pathway, providing important insights into the contribution of autophagy defects to the neurological phenotype of FD (87). Additionally, autophagy dysfunction has been implicated as a central mechanism in renal tubulointerstitial fibrosis in FD models (88). The role of impaired autophagy has also been examined in relation to corneal complications, highlighting its widespread impact on multiple organ systems (83).

### 5.5 Endoplasmic reticulum stress and the unfolded protein response

Recent studies have highlighted the important role of endoplasmic reticulum (ER) stress and the unfolded protein response (UPR) in the pathogenesis of FD, particularly in patients carrying missense GLA variants (9, 89). During biosynthesis, newly synthesized α-Gal A undergoes folding and post-translational modification within the ER before being transported through the secretory pathway to lysosomes. However, certain missense mutations may lead to protein misfolding and abnormal intracellular retention of α-Gal A within the ER and the endoplasmic reticulum–Golgi intermediate compartment (ERGIC), thereby disrupting the normal secretory pathway and triggering chronic ER stress and UPR activation. Unlike the classic enzymatic deficiency model of FD, in which disease manifestations are mainly attributed to glycosphingolipid accumulation within lysosomes, this mechanism suggests that cellular dysfunction may also arise independently of lysosomal storage (9, 89).

This mechanism may help explain why some heterozygous female FD patients become symptomatic despite having normal or only mildly decreased α-Gal A activity (90). Since ER stress and UPR activation arise from intracellular retention and misprocessing of mutant α-Gal A, wild-type α-Gal A secreted from cells expressing the normal GLA allele may be insufficient to reverse these intracellular pathological processes in cells expressing the mutated allele (9).

ER stress and UPR may also explain the variable therapeutic response observed in some FD patients carrying missense variants, as enzyme replacement therapy primarily compensates for enzymatic deficiency but may not correct the ER stress induced by misfolded α-Gal A proteins (91). Similarly, although migalastat may improve the stability and trafficking of certain mutant α-Gal A proteins, it may also enhance intracellular stress and UPR activation in some susceptible patients (92).

### 5.6 Other pathogenic pathways

In addition to disrupting the functions of intracellular organelles, Gb3 accumulation appears to influence the structure of the cell membrane as well. Birket et al. (2019) reported that FD cardiomyocytes derived from induced pluripotent stem cells exhibited increased sodium and calcium channel activity, leading to larger but shorter spontaneous action potentials (93). These observations are consistent with similar findings in neuronal ion channels, indicating that the buildup of Gb3 may modify ion channel expression or membrane trafficking, thereby affecting the electrical behavior of cells (94).

## 6 Vasculopathy in FD

FD-related vasculopathy can facilitate the development of accelerated atherosclerosis. This tendency may become more pronounced in the presence of comorbidities such as hypertension, renal dysfunction, or diabetes (95).

Gb3 disposition in endothelial cells and smooth muscle cells (SMCs) may be the earliest and the most prominent feature in FD, exerting harmful effects including vascular occlusion, luminal obstruction, contributing to ischemic tissue damage, disruption of the balance between vasodilators and vasoconstrictors, and thromboembolic complications (96, 97). Gb3 preferentially accumulates in VSMCs rather than endothelial cells, leading to VSMC proliferation and neointimal-like medial thickening. This process is characterized by inflammatory cell infiltration, increased intima-media thickness (IMT) and the formation of predominantly fibrotic rather than lipid-rich atheromatous lesions (98).

IMT in FD features in rare plaque formation and diffused location of stenotic lesions with different aspect (99). In the plasma of patients with FD, studies have identified growth-promoting factors, such as sphingosine-1-phosphate (S1P), that stimulate the proliferation of vascular smooth muscle cells and cardiomyocytes (97, 100, 101). Several studies have reported that plasma growth-promoting factors can increase IMT independently of Gb3 accumulation and lead to complications such as ischemia (102–104). The markedly elevated circulating Gb3 levels observed in FD patients have been shown to contribute to vascular structural alterations and to foster a prothrombotic endothelial state, thereby predisposing patients to a higher recurrence rate of ischemic events (105). Ischemia has a broad impact on the disease phenotype, indicating a systemic vasculopathy, involving small vessels in the heart, kidneys, peripheral nervous system, cerebrovascular system and skin (106).

Hemodynamic stress acting on a less compliant vascular wall may induce activation of the local renin–angiotensin system, with angiotensin II promoting inflammation through AT1-mediated upregulation of adhesion molecules, cytokines, chemokines, and ROS. These downstream effects enhance NF-κB activation and integrin expression, contributing to extracellular matrix expansion and vascular remodeling. Increased oxidative stress further reduces NO bioavailability and contributes to endothelial dysfunction (99). Moreover, Gb3 promotes cyclooxygenase-2 (COX-2) upregulation, enhancing endothelial inflammation and ROS production. This creates a positive feedback loop that further exacerbates endothelial dysfunction. However, COX-2–derived prostaglandins may partially offset the reduction in NO bioavailability (107). These inflammatory cascades promote protease-mediated extracellular matrix degradation and smooth muscle cell apoptosis, ultimately weakening the aortic wall and predisposing to aneurysm formation (108).

## 7 Cardiovascular involvement

Cardiovascular involvement is a major contributor to morbidity and mortality in FD. Clinically, patients often develop left ventricular hypertrophy (LVH), heart failure, and various arrhythmias (109). LVH is the most common structural cardiac abnormality in FD (110). The classic cardiac phenotype manifests as concentric hypertrophic cardiomyopathy, commonly referred to as Fabry cardiomyopathy (FC). This presentation is most frequently seen in middle-aged male patients. LVH in FD is primarily driven by Gb3 intracellular accumulation, hypertrophy-inducing growth factors, and extracellular matrix remodeling. Other factors, such as hypertension, aortic valve disease or cardiac amyloidosis, may further exacerbate left ventricular (LV) remodeling and hypertrophy (111).

In FD, Gb3 accumulates in multiple cardiac cell types, including cardiomyocytes, valvular fibroblasts, Purkinje fibers and nodal cells of the conduction system, vascular endothelial cells, and vascular smooth muscle cells. Gb3 deposition accounts for approximately 1%−2% of the total cardiac mass (112). Intracellular substrate accumulation contributes to progressive thickening of the cardiac walls and valve leaflet, producing LVH that may clinically resemble hypertrophic cardiomyopathy (HCM) (113). LVH progression and worsening diastolic dysfunction can lead to secondary cardiac structural remodeling, resulting in atrial enlargement and atrial fibrillation. In addition, deposition within intramyocardial vessel walls induces structural and functional vascular alterations, promoting ischemia and the development of replacement fibrosis, which may ultimately lead to systolic dysfunction (114).

LVH in FD is associated with multiple pathophysiological effects. Linhart et al. (2007) reported an inverse relationship between LV mass and estimated glomerular filtration rate (eGFR), and Barbey et al. (2006) reported a positive correlation between LV mass and the common carotid IMT (110, 115). Furthermore, LVH has been associated with a higher frequency of cardiac dysrhythmias (113).

In most FD patients, LV volume, LV dimension, stroke volume and LV ejection fraction (LVEF) are generally normal. Hypertrophy of the left ventricle and papillary muscles can reduce LV cavity size and contribute to left ventricular outflow tract (LVOTO) or mid-cavity obstruction (MCO), thereby aggravating symptoms such as chest pain, dyspnea, and syncope (116). While resting LVOTO is rare, it can be provoked in approximately half of patients with LVH (117).

Right ventricular (RV) involvement is also frequent in FD, and is primarily characterized by right ventricular hypertrophy (RVH). Although RVH correlates with overall disease severity and the extent of LVH, overt right ventricular systolic dysfunction is uncommon. Notably, patients with FD generally exhibit better right ventricular systolic performance than those with cardiac amyloidosis despite having similar degrees of right ventricular wall thickening (118).

Patients with FD may exhibit reduced exercise tolerance and exertional dyspnoea. These symptoms most commonly result from diastolic dysfunction, which can precede the development of LVH (119).

Diastolic dysfunction also leads to left atrial enlargement, thereby increasing susceptibility to atrial arrhythmias such as atrial fibrillation (113). Systolic dysfunction is less common in FD, with a prevalence of 6%-8%. Studies also shows that left ventricular systolic dysfunction is associated with increased mortality from heart failure (113).

FD–related cardiac manifestations can also include valvular involvement. Gb3 accumulates in both left- and right-sided cardiac valves, and patients with FD may develop valvular stenosis, regurgitation, and prolapse (120). Moreover, Gb3 accumulation, apoptosis, and vacuolation in the cardiac conduction system may cause autonomic dysfunction in FD, and are related to complications such as atrial and ventricular arrhythmias (121).

## 8 Renal involvement

As one of the most severe organ manifestations of FD, renal involvement frequently progresses to kidney failure and represents a major cause of both morbidity and mortality (122). The mechanisms include podocyte injury, microvascular disease, and tubulointerstitial injury (123).

Podocytes are terminally differentiated epithelial cells that play a central role in maintaining glomerular structure and the integrity of the filtration barrier (124). In FD, the degree of podocyte injury correlates closely with the severity of proteinuria and the progression of chronic kidney disease in FD (125). Because podocytes are postmitotic and possess minimal proliferative capacity, they cannot be replaced once lost through lethal injury. Works by Kriz et al. (1999) demonstrated that the denuded glomerular basement, resulting from podocyte depletion, contacts parietal epithelial cells and forms synechia, which ultimately leads to segmental glomerulosclerosis (126).

In individuals with the classic phenotype, Gb3 deposition in podocytes begins in intrauterine life (127). With advancing age, these deposits progressively expand within the podocyte cytoplasm, ultimately impairing podocyte integrity and function (123). Many studies have identified a positive correlation between podocyte Gb3 volume fraction (the proportion of the podocyte cytoplasm occupied by Gb3 inclusions) and urinary protein excretion rates (128, 129). The progressive accumulation of Gb3 may lead to podocyte foot process effacement, which is marked by the loss of normal interdigitations due to slit diaphragm disruption (130). Chronic podocyte injury increases mechanical strain and weakens podocyte adhesion to the glomerular basement membrane (GBM), eventually promoting their detachment (131). Glomerular hyperfiltration (HF) further increases biomechanical stress and elevates shear forces on foot processes, which in turn counteract their adhesion to the glomerular capillary wall (132). Abnormalities of the GBM contribute to podocyte loss and lead to significant podocyturia, which correlates with the severity of Fabry nephropathy (131, 133). Moreover, findings by Wise et al. (2025) indicate that ferroptosis-related mechanisms may contribute to podocyte damage in FD (134).

HF has been proposed as an early functional alteration in Fabry nephropathy and may serve as a potential early marker of renal involvement in FD (135, 136).

Riccio et al. (2018) suggested that HF may be a common feature in young FD patients (136). Previous studies have demonstrated that hyperfiltration in FD is associated with peripheral vascular alterations, including reduced arterial stiffness and endothelial dysfunction. These findings suggest that HF may reflect generalized microvascular and macrovascular functional abnormalities rather than an isolated renal hemodynamic change (137). Such vascular alterations may contribute to the development of renal hemodynamic abnormalities during the early stage of Fabry nephropathy.

Gubler et al. (1978) suggested that, the progressive glomerular and tubulointerstitial lesions in FD may be driven by ischemic injury (138). Fabry nephropathy is characterized by glomerulosclerosis with GBM wrinkling and partial collapse, along with tubular atrophy, interstitial fibrosis, and vascular wall thickening (139). These pathological changes are typically absent or only mild in patients younger than 25 years. These pathological changes result from Gb3 accumulation within vascular SMCs, leading to SMC necrosis and subsequent vascular compromise (123). Gb3 accumulation also occurs in tubular epithelial cells, contributing to focal tubular atrophy and interstitial fibrosis.

Jeon et al. (2015) demonstrated *in vitro* that Gb3 exposure may induce epithelial–mesenchymal transition (EMT) in human proximal tubular epithelial cells through upregulation of transforming growth factor-β (TGF-β) (140). Renal biopsy studies from FD patients further revealed increased TGF-β production in proximal tubular cells, which may activate myofibroblasts within glomerular and vascular compartments and promote tissue fibrosis (141). Accumulation of Gb3 may also activate the Notch1 signaling pathway and downstream NF-κB signaling, thereby enhancing chemokine production (142). Renal tubular cells may be damaged not only by intracellular accumulation of glycosphingolipids, but also by proteinuria-related toxicity. Exposure to excessive proteins in the ultrafiltrate, together with intracellular glycolipid deposits, may induce EMT in tubular epithelial cells, promoting the production of pro-inflammatory and pro-fibrotic cytokines and chemokines (143). These pathological processes may contribute to tubulointerstitial fibrosis in FD. Moreover, podocytes exposed to lyso-Gb3 produce TGF-β and upregulate extracellular matrix proteins through NOTCH1 and NF-κB activation, linking glycolipid accumulation directly to fibrogenic signaling (139).

These lesions reduce overall glomerular functional capacity. The remaining intact glomeruli may undergo compensatory hypertrophy, thereby predisposing to secondary focal segmental glomerulosclerosis (FSGS) (123) [a non-specific adaptive response commonly observed in chronic kidney diseases associated with nephron loss (144)].

Growing evidence indicates that FD pathogenesis extends far beyond simple Gb3 or lyso-Gb3 storage (139). Gb3 and lyso-Gb3 also activate inflammatory pathways. Leukocytes from FD patients exhibit increased adhesion molecule expression and constitutive release of pro-inflammatory cytokines (Interleukin-1, IL-6, and TNF-α), mediated by Gb3–Toll-like receptor 4 (TLR4) interactions on immune and renal cells (67). This TLR4-driven inflammatory activation promotes cross-talk between innate immunity and glomerular injury (145). Misfolded α-Gal A proteins may activate ER stress with UPR and contribute to kidney disease (9, 146).

. This mechanism may also be relevant to the variable renal response observed in some patients treated with migalastat, as enhanced transport of misfolded α-Gal A from the endoplasmic reticulum to the endoplasmic reticulum–Golgi intermediate compartment may further augment intracellular stress and UPR, potentially contributing to accelerated loss of kidney function (9, 92).

## 9 Central nervous system (CNS) involvement

In the central nervous system (CNS), vascular involvement in FD is well recognized, particularly affecting the vertebrobasilar and carotid arteries, with a higher prevalence in the posterior circulation (147). This can lead to a variety of neurological manifestations, including hemiparesis, vertigo or dizziness, diplopia, dysarthria, nystagmus, nausea or vomiting, headaches, hemiataxia, dysmetria, cerebellar gait ataxia, and, in rare cases, cerebral hemorrhage (147). Cerebral vasculopathy has also been associated with psychiatric symptoms and cognitive impairment (148). Epidemiological studies suggest that FD accounts for approximately 2–4% of strokes in individuals aged 18–55 years in the general population (149).

Numerous studies have documented structural CNS changes in FD (150). Brain lesions in FD tend to be diffuse (151). Cerebral atrophy has been observed in both gray and white matter in FD patients with no severe cerebrovascular disease. Region-specific atrophy—in the thalamus and hippocampus—has also been reported in FD (152). Additionally, reductions in overall intracranial volume have been noted, suggesting the possibility of altered neural development in FD (153).

In FD, Gb3 accumulation occurs in neurons of brain regions commonly implicated in neurodegenerative disorders. Gb3 deposits are primarily confined to neuronal somata, often exhibiting a swollen morphology, and are absent from dendrites, axons, and nuclei. Similar accumulations have also been observed in astrocytes, suggesting that CNS involvement in FD extends beyond cerebrovascular injury to include alterations in both neurons and glial cells (154). Deposits could occur in the dorsal motor nucleus of the vagus, substantia nigra, neocortex, and hippocampus (155). Studies have shown severe neuronal loss in the substantia nigra pars compacta accompanied by α-synuclein-positive Lewy pathology (154).

Growing evidence links FD to broader lysosomal dysfunction pathways that intersect with neurodegenerative and neuropsychiatric disorders. The psychiatric and cognitive manifestations of FD appear to arise from lysosomal and neuronal dysfunction rather than solely cerebrovascular injury (156).

Psychiatric and cognitive manifestations are increasingly recognized in FD. Depression is particularly common, with studies reporting high rates of depressive symptoms, anxiety, pain, and daytime sleepiness, and FD patients consistently scoring worse than the general population and patients with other chronic disorders (157). In a cohort study of 110 FD patients (60 heterozygous females and 50 hemizygous males), FD was linked to motor impairment and a range of nonmotor manifestations. The clinical pattern was not consistent with typical extrapyramidal symptoms, progressive cognitive impairment, or other prodromal manifestations commonly associated with neurodegenerative disorders such as Parkinson's disease or Dementia with Lewy bodies (158). Moreover, depressive symptoms do not correlate with stroke history or white matter lesions, implying that mood disturbances may arise independently of overt structural brain injury (159). Sleep disorders, including sleep-disordered breathing and periodic limb movements, are also prevalent and may exacerbate psychological burden (160).

In pediatric patients of FD, reduced quality of life has been documented, particularly among adolescents, due to psychological impacts of FD (159). Studies have also identified deficits in executive abilities, processing speed, and attention in FD. Ali et al. (2021) reinforces the presence of executive dysfunction in adults, with attention-deficit/hyperactivity disorder (ADHD)-like features reflected in difficulties sustaining attention and managing cognitive tasks (161).

## 10 Peripheral nerve involvement

Nerve involvements in FD include somatic and psychological impairment. The neurological manifestations primarily involve small-fiber neuropathy, which presents as pain, paresthesia, impaired thermal perception, and heat intolerance (162). Neuropathic pain is typically described as burning in a stocking-and-glove distribution, often accompanied by deep aches (163). Patients can also manifest gastrointestinal disturbances and pain crises (164). Symptoms mainly affect the distal extremities—including fingers, palms, and soles (163). Pain episodes can be triggered by heat and cold (165), and are associated with reduced tolerance to heat and physical exertion, impairing patients' quality of life (166).

Accumulation of Gb3 is considered one of the key contributors to peripheral neuropathic pain in FD. Deposits have been documented in the perineurium, endothelial cells of the vasa nervorum, dorsal root ganglia, and Schwann cells, along with associated ion-channel dysfunction within peripheral nerves (167). Pain crises in FD is attributed to Gb3 deposition within pain-sensitive dorsal root ganglion neurons (168). This storage disrupts normal nociceptive signaling, partly through alterations in ion channel function (167). In FD, accumulation of Gb3 and lyso-Gb3 has been also associated with chronic pain, likely mediated through transient receptor potential vanilloid (TRPV) channels as well as potassium, calcium, and sodium channels, which have been extensively investigated in DRGs of FD animal models (169).

Autonomic nervous system involvement is also common and may manifest as gastrointestinal disturbances resembling irritable bowel syndrome and as hypohidrosis (170). Abdominal pain and diarrhea are among the earliest and most frequent symptoms, while reduced sweating reflects selective injury to peripheral autonomic fibers (170).

## 11 Conclusion

Deficient α-Gal A activity caused by pathogenic *GLA* variants represents the primary molecular defect underlying FD. Mutations affecting the catalytic site, protein folding, intracellular trafficking, or post-transcriptional processing can reduce enzymatic activity to varying degrees, resulting in progressive accumulation of glycosphingolipids, predominantly Gb3 and lyso-Gb3, which represents the initiating event in the pathogenesis of FD. Variability in residual enzyme activity, together with factors such as XCI, epigenetic regulation, mutation-specific effects and UPR activation, contributes to the marked phenotypic heterogeneity observed among FD patients.

Pathological glycosphingolipid accumulation and subsequent lysosomal dysfunction may trigger a cascade of interconnected downstream events. Disruption of lysosomal homeostasis alters intracellular signaling and organelle crosstalk, contributing to dysregulation of mTOR signaling and impaired autophagic flux. Reduced mTOR activity and defective mitophagy promote the accumulation of dysfunctional mitochondria. Dysfunctional mitochondria with structural abnormalities result in impaired mitochondrial respiration, ATP depletion, and OXPHOS uncoupling. These alterations increase ROS generation while reducing antioxidant defenses, thereby promoting oxidative stress. Excessive ROS and ATP depletion may further aggravate lysosomal dysfunction by impairing lysosomal acidification, establishing a self-amplifying cycle between mitochondrial dysfunction and lysosomal impairment. Simultaneously, pathological lysosomal deposits may function as DAMPs, initiating inflammatory cascades characterized by cytokine release, immune dysregulation, and endothelial activation. Oxidative stress and inflammation further reinforce one another through positive feedback interactions, while accumulation of metabolic intermediates such as succinate may additionally amplify inflammatory signaling. Moreover, misfolded α-Gal A proteins may induce chronic ER stress and UPR activation, representing a pathogenic mechanism that may occur independently of lysosomal storage. Persistent crosstalk among lysosomal dysfunction, mitochondrial impairment, autophagic defects, oxidative stress, and inflammation ultimately contributes to vascular remodeling, fibrosis, ischemia, and progressive multi-organ injury in FD.

In the vasculature, endothelial dysfunction, oxidative stress, and chronic inflammation promote vascular smooth muscle cell proliferation, increased intima-media thickness, extracellular matrix remodeling, and prothrombotic changes, resulting in systemic vasculopathy and ischemic susceptibility. In the heart, intracellular Gb3 accumulation together with mitochondrial dysfunction, fibrosis, and microvascular ischemia contributes to ventricular hypertrophy, arrhythmias, and heart failure. In the kidney, glycolipid deposition within podocytes and tubular cells, combined with inflammatory signaling, ER stress, and autophagy defects, promotes proteinuria, tubulointerstitial fibrosis, and progressive renal dysfunction. Within the nervous system, vascular abnormalities, neuronal lysosomal dysfunction, and altered cellular stress responses contribute to stroke, neurodegeneration, cognitive manifestations, and small-fiber neuropathy. Collectively, these observations suggest that FD should be considered a systemic disorder driven by an integrated pathogenic network rather than a disease solely caused by lysosomal substrate accumulation.

Comprehensive understanding of these mechanisms is essential for early diagnosis, precise phenotypic stratification, and the development of targeted therapeutic strategies. Future research integrating genetic, metabolic, and molecular insights will be crucial to improve clinical outcomes and personalize treatment approaches in FD.
