# Supplementary material for: Pathogenic mechanisms in Fabry disease
Source: Front Med (Lausanne). 2026 Jun 23;13:1867822. doi: 10.3389/fmed.2026.1867822 (PMC13337472; doi:10.3389/fmed.2026.1867822)
Supplement: Supplementary file 1 [file Table_1.docx]

Supplementary Material

# Supplementary Figures and Tables

Supplementary Table 1

| **Biomarkers/ Mediators** | **Related Cells** | **Pathogenic Mechanism** | **Clinical Manifestation** |
| --- | --- | --- | --- |
| **Gb3 / Lyso-Gb3** | Endothelial Cells (ECs）, SMCs | Direct intracellular accumulation; triggers inflammation; disrupts NO synthesis | Endothelial dysfunction, oxidative stress, multi-organ involvement |
| **eNOS** | ECs | Enzyme uncoupling leading to NO reduction and ROS elevating | Impaired vasodilation, endothelial damage, elevated thrombosis risk |
| **ROS** | ECs, SMCs, neutrophils | Promotes oxidative injury and chronic inflammation | Tissue damage, oxidative stress–related injury |
| **ICAM-1 / VCAM-1** | ECs, leukocytes | Enhances leukocyte adhesion and vascular infiltration | Endothelial inflammation and progressive vascular injury |
| **TNF-α / IL-6** | Macrophages, T cells, ECs, SMCs | Pro-inflammatory cytokine signaling | Increased cardiovascular risk, inflammation-driven disease progression |
| **IL-10** | Macrophages, T cells, ECs | Anti-inflammatory cytokine; modulates immune response | Reflects attempts to counterbalance inflammation |
| **C3a / C5a** | Macrophages, neutrophils | Complement activation; promotes immune cell recruitment | Renal inflammation and endothelial injury |
| **C-reactive protein (CRP)** | Macrophages, hepatocytes, ECs, SMCs, lymphocytes | Acute-phase response; reduces NO activity; amplifies cytokine production | Indicator of vascular injury and systemic inflammation |
| **Vascular Endothelial Growth Factor (VEGF)** | ECs | Endothelial mitogen; stimulates angiogenesis | Associated with endothelial instability and disease severity |
| **Fibroblast Growth Factor 2 (FGF2)** | Fibroblasts, ECs | Modulates angiogenesis, cell proliferation, and tissue repair | Myocardial fibrosis, adverse cardiovascular events |
| **Transforming Growth Factor Beta 1 (TGF-β1)** | Macrophages, fibroblasts, ECs | Stimulates ECM synthesis and fibrosis pathways | Renal and cardiac fibrosis, LVH, disease progression |
| **Growth Differentiation Factor 15 (GDF-15)** | Macrophages, ECs | Stress-response mediator; regulates injury and fibrosis | Indicator of renal/cardiac injury and disease severity |
| **Myeloperoxidase (MPO)** | Neutrophils | Oxidizes LDL; reduces NO; activates MMPs | Vascular inflammation, atherosclerosis, coronary stenosis |
| **Syndecan-1** | ECs | Reflects glycocalyx shedding and endothelial barrier disruption | Correlates with cardiac and renal involvement |
| **MMP-2 / MMP-9** | Macrophages, fibroblasts | ECM degradation; remodeling pathways | Cardiac and renal fibrosis; vascular structural damage |

## Key inflammatory biomarkers and mediators
